# Effectively incorporating selected multimedia content into medical publications

**DOI:** 10.1186/1741-7015-9-17

**Published:** 2011-02-17

**Authors:** Alexander Ziegler, Daniel Mietchen, Cornelius Faber, Wolfram von Hausen, Christoph Schöbel, Markus Sellerer, Andreas Ziegler

**Affiliations:** 1 Institut für Immungenetik, Charité-Universitätsmedizin Berlin, Freie Universität Berlin, Berlin, Germany; 2 Structural Brain Mapping Group, Klinik für Psychiatrie und Psychotherapie, Universitätsklinikum Jena, Jena, Germany; 3 Institut für Klinische Radiologie, Universitätsklinikum Münster, Münster, Germany; 4 Abteilung für Innere Medizin, Bundeswehrkrankenhaus Berlin, Berlin, Germany; 5 Interdisziplinäres Schlafmedizinisches Zentrum, Charité-Universitätsmedizin Berlin, Berlin, Germany; 6 3D-Shape GmbH, Erlangen, Germany

## Abstract

Until fairly recently, medical publications have been handicapped by being restricted to non-electronic formats, effectively preventing the dissemination of complex audiovisual and three-dimensional data. However, authors and readers could significantly profit from advances in electronic publishing that permit the inclusion of multimedia content directly into an article. For the first time, the *de facto *gold standard for scientific publishing, the portable document format (PDF), is used here as a platform to embed a video and an audio sequence of patient data into a publication. Fully interactive three-dimensional models of a face and a schematic representation of a human brain are also part of this publication. We discuss the potential of this approach and its impact on the communication of scientific medical data, particularly with regard to electronic and open access publications. Finally, we emphasise how medical teaching can benefit from this new tool and comment on the future of medical publishing.

## Editorial note

During proofing and production of this article, there was significant debate about whether the multimedia files should be included as figures or additional files. We have taken the view that readers and indexers currently expect figures to be 2D graphical images suitable for printing, which is not the case with these files. The multimedia files are embedded into the PDF version of the article, and downloadable from links in the HTML version of the article. This article may act as a catalyst for Publishers to agree on the best way to present multimedia content.

## Introduction

Despite substantial improvements since the early attempts of anatomists during the Renaissance, medical illustrations have always been handicapped by being restricted to two dimensions (2D). Comparable, but even more severe limitations have prevented the distribution of moving images as well as sounds through medical publications. A common solution to communicate complex multimedia data currently relies on the creation of supplemental files that are available for download either through the publisher's or the author's website. However, this results in the unattractive separation of the actual publication from supporting multimedia data, which may contain crucial information (see [1] for an example). As electronic publishing gains momentum, it seems logical to fully exploit its potential by integrating multimedia and text files into a single article.

While online publishing formats are gaining popularity, there can be no doubt that the current standard in electronic publishing is the portable document format (PDF). Since June 2008, this file type provides also the possibility to integrate three-dimensional (3D), video, and audio content together with the text into a single file. Strangely, the potential of this technique does not seem to have been recognised so far in the field of medical publishing, while astronomers [2], chemists [3], structural biologists [4,5], as well as zoologists [6,7] have already exploited the many opportunities offered by this approach. Using examples from various medical disciplines, we demonstrate how the readers of medical publications can profit from embedded multimedia content. We also point out some of the opportunities that are now available for medical publishing in general.

## Interactive 3D imagery embedded into publications

To highlight the improvements that are attainable, we present here two fully interactive 3D models. The model of a face that is integrated into this article (Additional file 1) illustrates that the ability to freely manipulate a 3D structure, for example by zooming in on particular characteristics, offers interesting opportunities for a range of medical disciplines, since, for example, the shape of individual anatomical features of the skin could be apprehended by the reader in a truly interactive fashion. In contrast, the semischematic, surface-rendered 3D model of a human brain that is based on a magnetic resonance imaging dataset (Additional file 2) is intended to serve as an example of a greatly simplified anatomical representation of a human organ in 3D. In addition to colour coding, individual components have been labelled using so-called 3D mark-ups.

Interactive 3D imagery of human organs such as, for example, the brain is also available through the web (Table 1). However, most of the freely accessible brain models can be viewed only while the user is online, or require the installation of specialised software. In addition, certainly the greatest disadvantage of many of the models on offer is that they do not permit the viewer to 'take possession' of the visualisations and to disseminate them in any way desired.

**Table 1:** Websites offering interactive models of the human brain

Type of model	Website name	Web address
Free brains	3D Brain Anatomy	http://www.pbs.org/wnet/brain/3d/

	Allen Brain Atlas	http://www.brain-map.org

	Google Body Browser	http://bodybrowser.googlelabs.com

	Mapping Memory in 3D	http://ngm.nationalgeographic.com/2007/11/memory/brain-interactive

Paid brains	Anatomium 3D	http://www.anatomium.com

	Anatomy Planet	http://www.anatomyplanet.com

	Atlas of the Human Brain	http://www.thehumanbrain.info

	BrainNavigator	http://www.brainnav.com

	Visible Body	http://www.visiblebody.com

	Zygote	http://www.3dscience.com

In contrast, the advantages of the approach implemented here include (i) the full integration of the entire 3D model into the final publication, (ii) an intuitive, interactive form of access to the embedded model online as well as offline, (iii) the opportunity to disseminate the embedded model as desired, and (iv) the possibility to generate views and representations not predetermined by the author(s) of the manuscript, as would be the case, for example, in a video.

## Audiovisual content embedded into publications

The strength of videos, however, rests in their ability to convey time-dependent changes within an image, which is exemplified by a short video sequence embedded into this article (Additional file 3). Clearly, the possibility to integrate videos is likely to find a wide range of applications in medical publishing. As we show here, this is particularly evident in cardiology. Audio sequences can be integrated into a PDF as well. As an example, we present a striking case of sleep apnoea (Additional file 4). As in the case of videos, the integration of audio files into a publication might be of considerable interest for practitioners in several medical fields. For instance, documenting phenotypic variations in genetic disorders, such as in different variants of cri du chat syndrome [8], provides an example for the application of this technique. Readers not familiar with the use of PDF-embedded multimedia content will find a brief explanation towards the end of this article.

## Impact on medical publishing

The opportunities offered by embedding multimedia files have a number of foreseeable implications for publications that communicate medical data. From a scientific point of view, the greatest asset is presumably that the transparency of the presented data can be expected to increase, while the need for explanatory supplemental material will largely become obsolete. In recognising these opportunities, the *Journal of Neuroscience *has recently decided to ban supplemental material altogether [9]. Instead, authors are encouraged to 'publish articles with embedded movies or three-dimensional models, both online and in downloadable PDFs'. PDF files with embedded multimedia content are available as a single download and may be viewed online as well as offline at the reader's discretion. As stressed by the editor of the *Journal of Neuroscience*, this new policy 'eliminates the only essential role of supplemental material' and is meant to strengthen the desirable concept of an article as 'a complete, self-contained scientific report' [9].

We regard it also as important to point out that articles with embedded information are more likely to be written such that their multimedia content will be referred to as an integral part of the text (see, for example, [2-7]). Clearly, the implementation of such a reader-friendly approach to disseminating multimedia content will aid the transition from paper-based to pure electronic publishing. Having already stressed that publishers should adjust to publications with integrated multimedia content [10], we are confident that the acceptance of this novel format will be growing, as authors and publishing houses alike begin to realise its potential (see [5] for a number of examples). In our eyes, the universal acceptance of the PDF for the publication of scientific data guarantees a considerable life expectancy of this file format. We are confident that future software developments will take the existence of millions of PDF-based publications into account, providing future generations with the necessary backwards software compatibility.

Obviously, the form of communicating scientific results proposed by us is especially suited for electronic publishing, and in particular for those journals with an open access policy. In line with the entire philosophy behind the latter, all embedded multimedia content will become freely accessible over the web, resulting in the widespread dissemination of medical data such as those depicted here. In this scenario, limitations imposed by file size will no longer be an issue, since scientists can send each other a direct link to the article download site without the need to send individual papers by email.

The examples that we provide suggest that multiple medical disciplines will profit from the adoption of PDF-integrated 3D models or audiovisual content. However, a PDF-based scientific article must still be seen as a means to communicate scientific results in a reprocessed form, as opposed to the deposition of raw data in a database that permits a more comprehensive exploitation of this information, but requires also an advanced knowledge of computation or visualisation software tools [11].

## Didactic aspects

We would specifically like to point out the many opportunities that PDF embedding of multimedia files offers for the teaching of students. This pertains both to electronic textbooks as well as to lectures and seminars. In the case of interactive 3D imagery, the possibility to emphasise particular structures by labelling or colour coding is certainly an important feature (Additional file 2). In addition, a 3D model can be approached in two ways, either interactively (the user is always free to manipulate the object) or via a 'tour' (the author predetermines certain particularly instructive views of the object). The model tree in Additional file 2 provides an example for such a 'tour'. In addition to publications, PDF-embedded multimedia content is particularly well suited for presentations such as university teaching or conference talks.

## Medical publishing: *quo vadis*?

The current popularity of the PDF file format indicates that the option to embed multimedia content will persist for a considerable period. Clearly, the approach outlined in this article is relatively simple, but can greatly enhance the comprehension of medical publications, in particular those appearing in journals with purely electronic dissemination pathways. We expect that interdisciplinary research will also be facilitated by providing a universal platform for the exchange of scientific data and didactic material. However, precisely how technical developments in computation, visualisation, and archiving will influence medical publishing, both in offline and, increasingly, in online formats, is more difficult to foresee. For example, the recent advent of handheld devices that provide constant access to the internet as well as the availability of enhanced scientific publications [12,13] could significantly affect the way in which published medical data are apprehended in the future.

## Embedding multimedia content into PDF documents: a quick guide

Direct placement of multimedia content into a PDF file (Table 2 provides a list of supported file formats) can be accomplished using the commercially available Adobe Acrobat software (Adobe Systems, Mountain View, CA, USA) from version 9 onwards, as well as the freely available LaTeX package movie15 (available at http://www.ctan.org/tex-archive/macros/latex/contrib/movie15/). In the Adobe Acrobat software, multimedia files can be converted into a PDF using the 'multimedia tool' buttons. Using the 'selection' tool, PDF-embedded multimedia content can be extracted and copied into another PDF using conventional copy and paste keyboard combinations. The sizes of the individual multimedia files embedded into this article are 3.3 MB for the face (Additional file 1), 10.5 MB for the human brain (Additional file 2), 1.1 MB for the video (Additional file 3), and 2 MB for the audio sequence (Additional file 4).

**Table 2:** List of file formats that can be used in direct placement into portable document format (PDF) documents using Adobe Acrobat versions 9 and X

Multimedia type	File format or type	File extension
Audio	MP3 audio	mp3

Three-dimensional image	Autodesk 3D Studio	3ds

	Product Representation Compact (PRC)	prc

	Stereolithography	stl

	Universal 3D	u3d

	Virtual Reality Modelling Language	vrml, wrl

	Wavefront Object	obj

Video	Flash video	flv, f4v

	MPEG	mp4, m4v

	QuickTime Movie	mov

	Shockwave Flash	swf

The freely available LaTeX package movie15 http://www.ctan.org/tex-archive/macros/latex/contrib/movie15/ covers a similar range of file formats. The 3D file formats presented here constitute a selection of those available.

Viewing of PDF-embedded multimedia content requires the use of the freely available Adobe Reader version 9 (or onwards) on Windows, Macintosh, or Linux systems. Activation of the embedded multimedia file can be achieved by 'left clicking' anywhere on the additional file ('Click for 3D/Audio/Video').

With regard to embedded 3D models, their performance depends largely on the properties of the reader's computer system, in particular its graphics card capability and a sufficient availability of RAM. In order to interactively manipulate the embedded 3D elements (zoom, drag, toggle, and so on), it is necessary to use the mouse button(s). The toolbar (present in the top-left corner of the multimedia window that is seen after activation of the embedded 3D model) can be employed to change background colour as well as lighting. A model tree can be activated by 'left clicking' onto the respective icon to the right of the 'Views' drop-down menu. This model tree permits an access to individual substructures of the model and to switch their visibility as well as the transparency on or off. A 'tour' can be activated by clicking on the green arrows in the centre of the opened model tree menu. The interactive multimedia session can be terminated by 'right clicking' anywhere on the model and choosing 'Disable 3D'. A comprehensive description on how to create PDF-embedded 3D models can be found in [5,6], whereas [14] provides a more detailed explanation on how to manipulate interactive imagery.

## Competing interests

AlZ, DM, CF, WvH, CS, and AnZ declare that they have no competing interests. MS is an employee at 3D-Shape GmbH (Erlangen, Germany).

## Authors' contributions

AlZ and AnZ designed the study. AlZ and DM carried out automated and manual segmentation as well as PDF embedding. CF performed brain MRI. WvH carried out the ultrasound investigation. CS carried out audio and video recording. MS performed face scanning and PDF embedding. AlZ, DM, and AnZ prepared a first draft of the manuscript and all authors contributed to its writing and approved the final version.

## Pre-publication history

The pre-publication history for this paper can be accessed here:

http://www.biomedcentral.com/1741-7015/9/17/prepub

## Supplementary Material

Additional file 1 **Portable document format (PDF)-embedded, interactive three-dimensional (3D) model of a face**. The 3D model was generated using an optical face scanner (FaceSCAN^3D^, 3D-Shape GmbH, Erlangen, Germany). This system measures the 3D shape of an object in less than a second using projected light patterns and a set of cameras. Applications of this methodology may include, for example, before and after surgery comparisons and the documentation of dermatological or orthopaedic patient characteristics. Scanning was performed on a healthy male volunteer. Activation of the embedded multimedia content requires the use of a PDF reader compatible with version 1.7 Extension Level 3 (for example Adobe Reader 9). Use the '+/- zoom' or 'toggle full-screen' options in order to maximize window size. Click here for file

Additional file 2 **Portable document format (PDF)-embedded, interactive three-dimensional (3D) model of a human brain**. This 3D model is based on a magnetic resonance imaging (MRI) dataset from a healthy female volunteer which was acquired with 500 μm isotropic resolution using a 3D protocol (3DT1TFE) on a Philips Achieva 3 T scanner (Philips Healthcare, Eindhoven, The Netherlands). Segmentation and modelling were accomplished using automated brain extraction with the FMRIB software library (FSL) brain extraction tool (BET) [15], automated segmentation of the cortical grey matter based on the hidden Markov random field-expectation maximisation (HMRF-EM) framework by means of FSL FMRIB automated segmentation tool (FAST) [16], and manual segmentation of the remaining structures using Amira 5.2 (Visage Imaging GmbH, Berlin, Germany). Note the pronounced differences in surface mesh quality between the 2D cover image and the interactive model, which are a consequence of the need to reduce the final file size. FMRIB = The Oxford Centre for Functional MRI of the Brain. Activation of the embedded multimedia content requires the use of a PDF reader compatible with version 1.7 Extension Level 3 (for example Adobe Reader 9). Use the '+/- zoom' or 'toggle full-screen' options in order to maximize window size. Click here for file

Additional file 3 **Portable document format (PDF)-embedded video sequence of a human heart**. This 10 s long video sequence of the beating heart of a healthy male volunteer was obtained using a Philips iE33 xMATRIX echocardiography ultrasound system with colour flow and pulsed wave/continuous wave Doppler (Philips Healthcare, Eindhoven, The Netherlands). Activation of the embedded multimedia content requires the use of a PDF reader compatible with version 1.7 Extension Level 3 (for example Adobe Reader 9). Use the '+/- zoom' or 'toggle full-screen' options in order to maximize window size. Click here for file

Additional file 4 **Portable document format (PDF)-embedded audio sequence of a male patient suffering from severe sleep apnoea**. This audio sequence, with a duration of 47 s, begins with strong rhythmic snoring, followed by a long (32 s) period of sleep apnoea, before snoring is finally resumed (the slightly audible background noise is due to a TV set). Activation of the embedded multimedia content requires the use of a PDF reader compatible with version 1.7 Extension Level 3 (for example Adobe Reader 9). Click here for file
