# Supplementary figures and images for: Effectively incorporating selected multimedia content into medical publications
Source: BMC Med. 2011 Feb 17;9:17. doi: 10.1186/1741-7015-9-17 (PMC3040697; doi:10.1186/1741-7015-9-17)

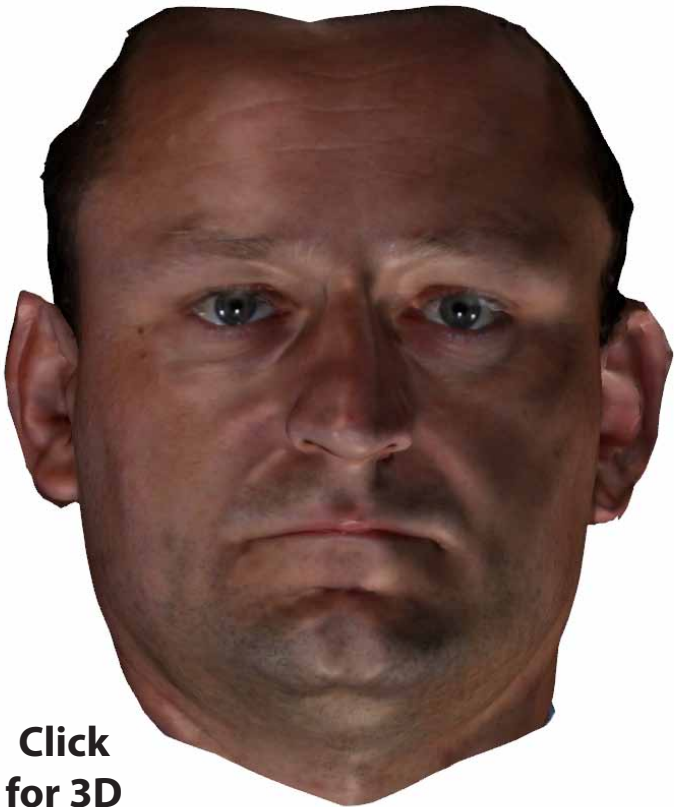

**Click  
for 3D**

Supplement: Additional file 1 — Portable document format (PDF)-embedded, interactive three-dimensional (3D) model of a face. The 3D model was generated using an optical face scanner (FaceSCAN3D, 3D-Shape GmbH, Erlangen, Germany). This system measures the 3D shape of an object in less than a second using projected light patterns and a set of cameras. Applications of this methodology may include, for example, before and after surgery comparisons and the documentation of dermatological or orthopaedic patient characteristics. Scanning was performed on a healthy male volunteer. Activation of the embedded multimedia content requires the use of a PDF reader compatible with version 1.7 Extension Level 3 (for example Adobe Reader 9). Use the '+/- zoom' or 'toggle full-screen' options in order to maximize window size. [file 1741-7015-9-17-S1.pdf]

**Click  
for 3D**

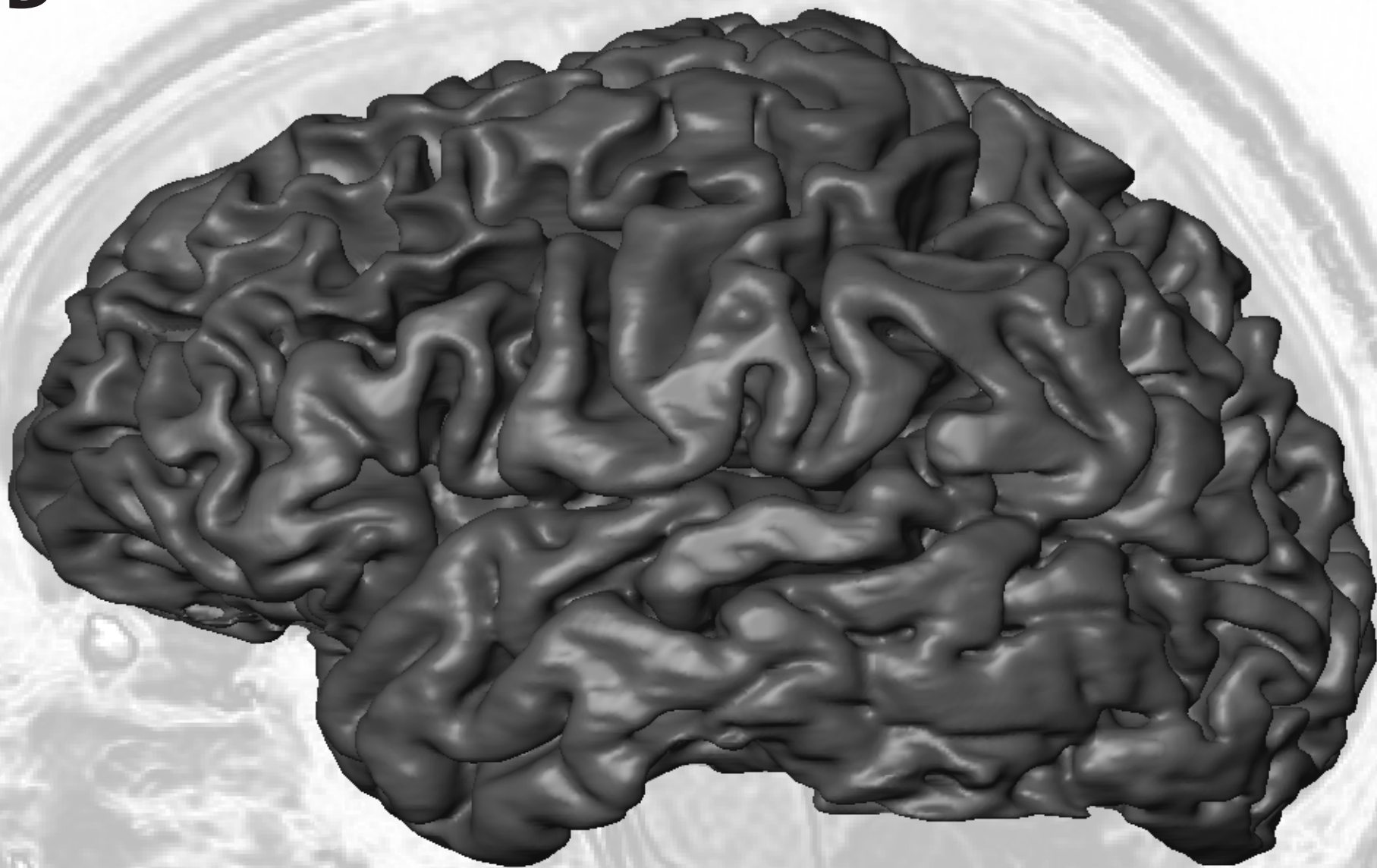

Supplement: Additional file 2 — Portable document format (PDF)-embedded, interactive three-dimensional (3D) model of a human brain. This 3D model is based on a magnetic resonance imaging (MRI) dataset from a healthy female volunteer which was acquired with 500 μm isotropic resolution using a 3D protocol (3DT1TFE) on a Philips Achieva 3 T scanner (Philips Healthcare, Eindhoven, The Netherlands). Segmentation and modelling were accomplished using automated brain extraction with the FMRIB software library (FSL) brain extraction tool (BET) [15], automated segmentation of the cortical grey matter based on the hidden Markov random field-expectation maximisation (HMRF-EM) framework by means of FSL FMRIB automated segmentation tool (FAST) [16], and manual segmentation of the remaining structures using Amira 5.2 (Visage Imaging GmbH, Berlin, Germany). Note the pronounced differences in surface mesh quality between the 2D cover image and the interactive model, which are a consequence of the need to reduce the final file size. FMRIB = The Oxford Centre for Functional MRI of the Brain. Activation of the embedded multimedia content requires the use of a PDF reader compatible with version 1.7 Extension Level 3 (for example Adobe Reader 9). Use the '+/- zoom' or 'toggle full-screen' options in order to maximize window size. [file 1741-7015-9-17-S2.pdf]

**BF 22Hz**  
15cm

**2D**  
75%  
K 50  
M Niedrig  
HAllg

**FD**  
66%  
2.5MHz  
WF Hoch  
Mittel

S3 S4  
+61.6  
-61.6  
cm/s

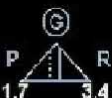

**Click  
for Video**

JPEG

64 /min

Supplement: Additional file 3 — Portable document format (PDF)-embedded video sequence of a human heart. This 10 s long video sequence of the beating heart of a healthy male volunteer was obtained using a Philips iE33 xMATRIX echocardiography ultrasound system with colour flow and pulsed wave/continuous wave Doppler (Philips Healthcare, Eindhoven, The Netherlands). Activation of the embedded multimedia content requires the use of a PDF reader compatible with version 1.7 Extension Level 3 (for example Adobe Reader 9). Use the '+/- zoom' or 'toggle full-screen' options in order to maximize window size. [file 1741-7015-9-17-S3.pdf]

**Click  
for Audio**

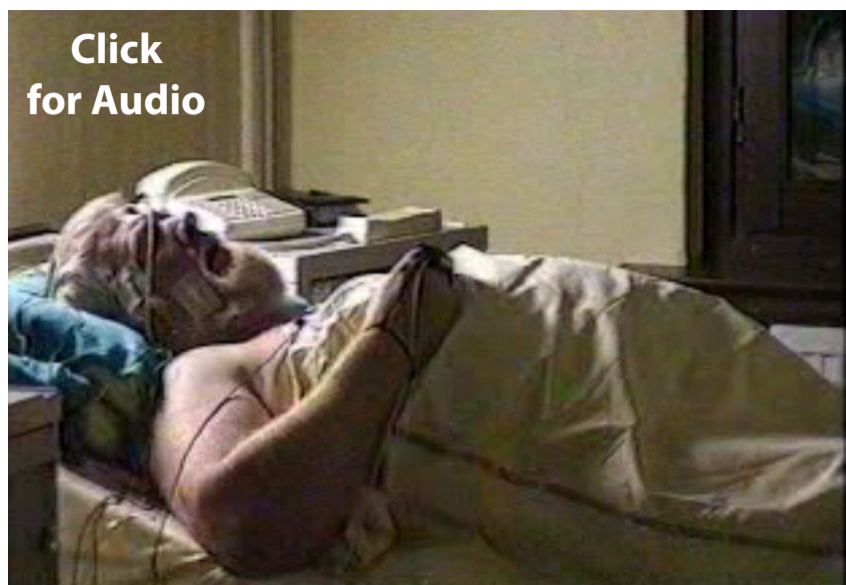

Supplement: Additional file 4 — Portable document format (PDF)-embedded audio sequence of a male patient suffering from severe sleep apnoea. This audio sequence, with a duration of 47 s, begins with strong rhythmic snoring, followed by a long (32 s) period of sleep apnoea, before snoring is finally resumed (the slightly audible background noise is due to a TV set). Activation of the embedded multimedia content requires the use of a PDF reader compatible with version 1.7 Extension Level 3 (for example Adobe Reader 9). [file 1741-7015-9-17-S4.pdf]
